# Private or salaried practice: how do young general practitioners make their career choice? A qualitative study

**DOI:** 10.1186/s12909-016-0754-6

**Published:** 2016-09-01

**Authors:** Shérazade Kinouani, Gary Boukhors, Baptiste Luaces, William Durieux, Jean-Sébastien Cadwallader, Isabelle Aubin-Auger, Bernard Gay

**Affiliations:** 1 Univ. Bordeaux, UMR1219, F-33000 Bordeaux, France; 2 INSERM, team HEALTHY, UMR1219, F-33000 Bordeaux, France; 3 Department of General Practice, Univ. Bordeaux, F-33000 Bordeaux, France; 4 Department of General Practice, Sorbonne Universités, UPMC Univ Paris 06, School of Medicine, F-75012 Paris, France; 5 Department of General Practice, Sorbonne Paris Cité, Univ Paris Diderot, F-75018 Paris, France; 6 EA Recherche Clinique Coordonnée Ville-Hôpital, Méthodologies et Société (REMES), F-75018 Paris, France; 7 Département de Médecine générale, Université de Bordeaux, 146, rue Léo Saignat, Case 148, 33076 Bordeaux, Cedex France

**Keywords:** Career choice, General practitioners, Primary health care, Fee for service, Salary, Medical education, Professional practice location, Qualitative research

## Abstract

**Background:**

Young French postgraduates in general practice increasingly prefer salaried practice to private practice in spite of the financial incentives offered by the French government or local communities to encourage the latter. This study aimed to explore the determinants of choice between private or salaried practice among young general practitioners.

**Methods:**

A qualitative study was conducted in the South West of France. Semi-structured interviews of young general practitioners were audio-recorded until data saturation. Recordings were transcribed and then analyzed according to Grounded Theory by three researchers working independently.

**Results:**

Sixteen general practitioners participated in this study. For salaried and private doctors, the main factors governing their choice were occupational factors: working conditions, need of varied scope of practice, quality of the doctor-patient relationship or career flexibility. Other factors such as postgraduate training, having worked as a locum or self-interest were also determining. Young general practitioners all expected a work-life balance. The fee-for-service scheme or home visits may have discouraged young general practitioners from choosing private practice.

**Conclusions:**

National health policies should increase the attractiveness of ambulatory general practice by promoting the diversification of modes of remuneration and encouraging the organization of group exercises in multidisciplinary medical homes and community health centers.

## Background

France is one of the European countries where group practice concerns only a minority of general practitioners (GPs). In 2003, it concerned only 39 % of French GPs while over 90 % were concerned in Sweden, Finland, Canada or United Kingdom [1]. The main type of group practice in France is private. In fact, the French primary health care system is widely based on private general practice with GPs working alone or in a group. As in Belgium and Germany, the fee for service (FFS) is the main mode of remuneration of these French GPs [1]. This means that a part of the fees for each consultation or home visit are paid by the patient and the rest by the national health insurance fund.

In January 2015, there were 89788 GPs in France of whom 65 % were in private practice [2]. Previous studies showed that young French postgraduates in general practice increasingly prefer salaried practice to private practice [2–4]. The number of private GPs has decreased by 4.4 % these last 7 years, while the number of salaried GPs has increased by 4.5 % [3]. These trends could continue until 2020 in spite of several measures taken by the French government and local communities over the last two decades to encourage young doctors to choose private practice [5]. These measures include increasing the duration of the general practice training of medical students, facilitating the implementation of multidisciplinary medical homes in underserved areas, and especially providing national tax breaks or financial incentives from local communities. The effect of the latter measures remains unclear and under-assessed. Using financial incentives to encourage doctors to practice in underserved areas has already been tested in other countries. Although the issue has received little attention, the measure is thought have a small positive effect [6, 7].

Several studies have examined the factors that influence how medical students choose to become GPs [8–14]. Some have shown that students do not always know at the beginning of their medical education which specialty they would like to practice, some of them changing their minds during their training [15–19]. We considered it would more appropriate to ask young GPs what might have influenced their choice. Few studies to date have focused on factors which explained the choice between private and salaried practice [20–22] and this issue has not received any attention in France. Any regulatory policy impacting medical demography should take into account the expectations of young GPs and the feminization of the medical occupations. Otherwise, this would be akin to ignoring the changes taking place in society and within these occupations.

The aim of this study was therefore to explore the determinants of choice between private or salaried practice among young GPs in France.

## Methods

Face-to-face semi-structured interviews were conducted between June 2013 and April 2014 with 16 young GPs in Gironde (France) according to a Grounded Theory approach. The interviews were conducted on a one-to-one basis to allow personal opinions to be freely expressed since the GPs may have had personal reasons to explain their career choice that they preferred to discuss in private. Moreover, they may have had radically opposing views on the subject so it was important to avoid a confrontational atmosphere.

### Participants

To be eligible for the study, GPs had to work in a private establishment or have a salaried medical occupation. They had to have obtained their postgraduate degrees between September 1st 2002 and December 31st 2012, i.e. less than 11 years before the beginning of the study. Locum GPs were excluded.

A purposive sample of GPs was selected in order to obtain a maximum diversity of experiences and opinions. The following information was gathered: practice (private or salaried), gender, practice location (urban or rural area) and declaration (or not) of having had additional training to general practice (for example: alternative medicine, emergency medicine, pediatrics, gynecology, etc.). GPs were considered to have a private practice when more than 75 % of their occupational time was spent in a private establishment. They were considered salaried if they declared more than 75 % of their time in salaried occupations. Urban areas were defined as communities of 10,000 inhabitants or more.

### Recruitment stage

One of the authors (GB) called the “Conseil Départemental de Gironde” to ask for help in recruiting salaried GPs who worked in their various services. This authority is responsible for local services such as Maternal and Child Protections or Departmental Homes for Disabled Persons. GB also called the management of the University Hospital of Bordeaux and two health insurance funds in Gironde: “Caisse Primaire d’Assurance Maladie” and “Mutualité Sociale Agricole”. All these authorities agreed to help in recruitment. They sent a mail advert to GPs corresponding to the required profiles or sent us the addresses and phone numbers of departments in which eligible employees worked. GB contacted private GPs through the French phone directory. An advert was also sent by mail to all young GPs and locum GPs who were in a regional online forum.

All doctors were approached by phone to verify their eligibility. None of them refused to participate. GB set up meetings with the GPs for the semi-structured interview. At the end of each interview, the respondents were asked if they knew other young GPs who could participate in the study. Thus, the sample accrued by a snowball effect.

### Data collection

The topic guide was initially designed from data in the literature. It was tested in two individual interviews before being used for the study and then was modified. The final topic guide is shown in Table 1.

**Table 1:** Final topic guide

Questions	Themes
Icebreaker question	Would you describe yourself briefly? →Age, gender, number of years of practice, etc.
Reasons for choice of private (or salaried) practice	*Occupational criteria* →Further training in general practice →Expected income →Fee for services →Continuity of care →Occupational autonomy →Pace of work →Administrative tasks →Relationship with peers →Opportunities for setting up practice →Proximity of medical and paramedical infrastructure, care networks *General practice characteristics* →Representation of current general practice →Representation of general practice in the future *Postgraduate training in general practice* →Training in private general practice *Role of GP locum* *Personal factors* →Parenthood project →Social protection →Free time →Time spent with family →Area of origin →Family history
Reasons for not choosing private (or salaried) practice	Summarise themes above
Career perspective	What do you think of the choice you made? →Regrets →Desire to change type of exercise later
Knowledge of GPs about installation incentives	→Financial incentives in underserved areas
Mixed practice	→Benefits and disadvantages →Mixed practice and diversity of practice settings →Mixed practice and quality of care
Community health centers	→Knowledge about community health centers →Representation of community health centers
Other aspects that interviewees want to discuss	

The interviews were conducted by GB. The meeting place was chosen by the interviewee to create a reassuring atmosphere. Most interviews were conducted in private and salaried GPs’ offices. One of the interviews took place in a café. Their agreement to participate was requested a second time just before recording began.

The interviews had to be continued until data saturation, until there were no longer any new hypotheses during the interviews. Saturation seemed to be obtained with the fourteenth interview. Each interview was recorded on audiotape and later transcribed by GB.

### Data analysis

The data analysis was performed concurrently with data collection according to the Grounded Theory approach. This allowed a constant comparison between collected data and analyzed data and to identify new hypotheses. The first three authors (SK, GB and BL) performed the analysis in four steps. They were all trained in qualitative research. First, each author independently broke the transcripts down into words or sentences to obtain open codes. Second, the open codes were classified into subcategories or axial codes. Third, the subcategories were classed into themes. The fourth step was to use the thematic analysis to develop a theory about the factors determining the choice between private and salaried practice.

Open coding was performed by the three researchers by using NVivo 8® software (QSR International, 2008, Canada). Axial coding was performed by one of us (SK) using mind mapping with Freemind 1.0 ® software. The other authors (GB, BL) used NVivo 8 ® for axial coding. At each step, the three authors compared their analyses. This method of triangulation aimed to validate the similarities or to obtain a consensus about their discrepancies of interpretation.

### Ethics and consent

According to French law, it was not necessary to submit the protocol to an ethics committee. Therefore, we did not seek the advice of the local privacy protection committee for this study. Oral consent to participate in the semi-structured interviews was collected by telephone. Before each interview, all GPs received a letter reminding them that a meeting was planned, that the interview would be recorded and that the transcripts would be anonymized. It also stipulated that a financial compensation was offered (23€) if they still agreed to participate. The letter also briefly presented the first two authors. They had to sign a form attached about their incentives to the letter and give it to the interviewer on the day of the meeting.

## Results

Sixteen young GPs (age range 30 to 40 years) were interviewed in this study (8 men, 8 women). An additional file shows all interviews in more detail. This is available on request to the corresponding author. Meetings lasted between 20 and 45 min. Their characteristics are shown in Table 2.

**Table 2:** Characteristics of participants

Factors used for purposive sampling			
Location practice	Additional trainings to general practice	Choice of Practice	Gender
Urban	Yes	Salaried	2 men, 1 woman
		Private	1 man
Urban	No	Salaried	1 woman
		Private	1 man, 2 women
Rural	Yes	Salaried	2 men, 2 women
		Private	1 man, 1 woman
Rural	No	Private	1 man, 1 woman

Six main themes were identified: occupational factors, postgraduate training, having worked as a locum, general practice characteristics, personal factors and self-interest.

### Occupational factors

#### A guaranteed minimum income

Young GPs expected to earn a guaranteed minimum income. This concept may be understood in several ways. First, it was considered to be a fair return on their years spent in medical education. Second, for salaried GPs it meant receiving a known monthly income that brought with it reassurance and financial security. Third, some private GPs appreciated the possibility of adjusting their income according to their needs.

*For me, I consider that as a general practitioner, I should receive a guaranteed minimum wage. I did not see myself having to do 10 or 12 years of study to earn peanuts so … The condition for me was therefore salaried practice with approximately a fair wage* (participant 6, man, salaried GP)

*The freedom to choose just what we want to earn. By doing so and depending on our activity, we can approximately calculate our future income* (participant 11, woman, private GP)

Two young private GPs recognized that in private practice, their income might be higher than in a salaried hospital practice. However, a higher level of income was not seen as a priority.

Salaried GPs mentioned the FFS. They thought some GPs were made for this scheme and others were not. You had to be in agreement with it if you were a private GP. Being a salaried doctor without FFS was a guarantee of being able to prescribe freely. Some salaried GPs felt embarrassed about charging patients at the end of a consultation, especially if it had been short.

#### A varied scope of practice

Some GPs wanted to have a varied scope of practice and found it in either private or salaried practice.

*I did not want to limit myself to something special and I find that in general practice, finally, we see all kinds of people, we can be consulted for conventional or rare diseases* (participant 12, woman, private GP)

*If I get bored, my work no longer suits me. In fact, I need variety* (participant 2, man, salaried GP)

These GPs (both salaried and private) were disappointed by their previous experience in urban areas, which they found boring. One GP found this type of variety through mixed practice: he worked as a salaried doctor in several locations but had a private practice one day per week.

#### The relationship with patients

Private GPs reported searching for a closeness in their relationship with patients and they thought it was impossible to have it in a salaried practice. Such closeness counterbalanced their work difficulties. More than being a doctor for each patient, they were a family doctor. Closeness was seen to be part of rural practice.

Some salaried GPs reported a deterioration in the doctor-patient relationship. They found patients lacked recognition and respect today more than before. This was partly the consequence of medical outreach because of internet. Other salaried GPs found this relationship had evolved towards a provider-client relationship. Patients had requests that were more and more pressing and unjustified. They thought private GPs adapted to this trend by satisfying all requests made to them. Salaried GPs described this trend as meeting the client’s wishes and felt it was reinforced by the FFS. Indeed, a private GP could not systematically respond unfavorably to unjustified requests, otherwise his patients would eventually shop around for another GP because they would expect to have “value for money” when they paid for their consultation.

#### Autonomy

GPs enjoyed their autonomy in private general practice. They sometimes reported previous difficulties with a hierarchical superior who had been instrumental in their quitting a position as doctor.

*You want to pilot your career rather than be piloted by someone else (sigh). I had an experience in hospital that ended badly so I naturally turned to private practice* (participant 7, man, private GP)

#### Working conditions

This was a major factor combining several aspects: workload, working time, administrative tasks and teamwork. The workload was often described as a barrier to choosing private practice, especially in rural areas. The varied scope of general practice partly explained this workload. It was felt that private GPs should be able to manage complex care problems while responding rapidly to emergencies. In this context, time became a source of pressure on doctors.

*And then I was depressed in advance when I saw 30 planned appointments every quarter of an hour (sigh), I was depressed* (participant 10, woman, salaried GP)

*This is not the same pressure: in private practice, we have a number of patients, several patients waiting in the waiting room and we must … we cannot dwell on patients with complex problems, and it’s difficult to exceed the time limit. We have to see all the patients. In the Emergency Department, it is true that there is the pressure of time but it's not the same. If there is a critically ill patient, it is natural to spend more time on him so less critically ill patients have to wait* (participant 2, man, salaried GP)

Work schedules worried most young GPs. Two women chose medical employment to have more flexible and shorter hours. They wanted to spend more time with their children. This was more important than having a higher income. The need for working time flexibility was also reported by some private GPs. They thought they could reschedule their working time if necessary without having to answer to an employer and could finish earlier for family reasons. They could also take leave when they wished.

*In fact, what attracts me in private practice, it’s the freedom that we can have regarding the organization of our working time, for vacations, especially when you have children. For me, I had to have this freedom to take time off when I wanted at times coinciding with the school vacations* (participant 11, woman, private GP)

Salaried GPs were put off by the pace imposed by home visits. It could be a barrier for salaried GPs while private GPs liked them because they created a change in the work schedule.

Sometimes, working time flexibility was found with part-time work for both private and salaried GPs. They thought that the feminization of the health professions had contributed much to the overall decrease in working time and the attraction for part-time work. This change could be an asset to changing the health care system and working conditions in the coming decades.

*It's good. The great advantage I see is that it will force physicians to work differently. That will change some practices: it'll incite everybody to work in group practice, perhaps force us to reconsider continuity of care so that we might also be reachable with schedules organized differently. I think it's a good opportunity to seize on. Perhaps, reorganize primary care with more leeway given to nurses. Young female GPs will probably not want to work as physicians as they did 20 years ago. And this is perhaps just as well* (participant 7, man, private GP)

Administrative tasks were not considered a factor that influenced their choice but rather as a source of dissatisfaction for private GPs. They thought managing a medical office was time-consuming and stressful. They learned to do their accountancy on-the-job and during their work as a locum. However, administrative tasks remained a part of their job. Some GPs had found a way to delegate tasks to a management organization or an accountant.

According to salaried GPs, teamwork avoided isolation and promoted multidisciplinary care. For private GPs, teamwork could be found in group practices. It offered the chance of more free time or sharing medical office fees with peers.

#### Career opportunities

For some private GPs, career opportunities cropped up and were decisive. For one, the opportunity was the retirement of a GP who had a medical practice reflecting the young GP’s expectations. Another young GP currently worked with a GP that he previously had replaced. Another GP expected to have a job that never materialized so he chose private practice by default.

Some salaried and private GPs thought that they would not continue in their current practice all their lives and would change later. For all, their current practice offered career flexibility.

### Postgraduate training and having worked as a locum

All GPs reported that postgraduate clerkships were decisive in their choice of practice. These clerkships had confirmed or had overturned a choice. Sometimes, they had revealed the occupation that they had at present. Choosing a practice could also be determined by encounters with role models, with positive or negative effects.

*I did not want to do all emergency medicine when I was a student and I went to cardiology intensive care … My clerkship in emergency medicine, my duty periods, all this influenced my choice* (participant 2, man, salaried GP)

Having worked as a locum allowed some to form an opinion on private general practice. It had also helped to choose the location of their practice.

*After having worked as a locum for 10 years, I could see private practice in all its forms, both advantages and disadvantages. I saw how I liked to work, I liked private general practice a lot. I don't see myself working otherwise* (participant 4, man, private GP)

### General practice characteristics

Conformity of personal considerations about primary care with the characteristics of general practice had a strong influence on the choice of practice. Some had identified GPs as the first contact for patients within the healthcare system and as a health promoter. They described how GPs managed both acute and chronic problems and coordinated them. They spoke about GPs as being doctors who establish a relationship over time, provide continuity of care and integrate a social dimension into care. Private GPs were attracted to these characteristics while some salaried GPs sought to avoid them.

*Coaching patients, giving nutritional advice or advice for daily life, listening to their daily worries, it's true that this is not what I like* (participant 2, man, salaried GP)

### Personal factors

#### Work-life balance

Young GPs expected this. This balance partly depended on working time and required not returning home late. Some had chosen a salaried practice because their spouse was a private GP and they thought it would be more difficult if they were both private GPs. Others chose private general practice because they found it easier to monitor their working time being a private GP at home. The partner’s work (or opportunities to find it) also determined the practice location.

*What affects us all are the possibilities for our partners to find work. So I had a criterion: my wife already had a job in the big city. I could fit in with that, I had a radius of about 1:00 to 1:30 or thereabouts* (participant 5, man, salaried GP)

*I wanted to stay… My husband works here, in hospital. So, I wanted to stay in or around the city* (participant 12, woman, private GP)

#### Guarantee of social protection

Some salaried GPs recognized that they had been attracted by the better guarantees of social protection that being salaried in France carries, especially the risks of sickness and workplace accidents. Female GPs spoke about the risks of pregnancy and maternity. They thought that although there had been improvements in social protection with regard to these risks, the levels of risk were lower for salaried GPs.

Having a parent doctor could have a negative or positive effect on choice of practice. A salaried GP explained that if he had chosen private practice, he would have worked with his father. He wanted to avoid the situation where patients compared their practice. Another salaried GP remembered how his father, who had been a private GP, was frequently absent from home.

### Self-interest

This was frequently cited as a determining factor. GPs chose a type of practice they thought it would be more intellectually stimulating or less monotonous. They also thought that this was a way to gain new knowledge and skills. Sometimes, general practice was a default choice, self-interest arising later.

Some GPs also thought the choice could be governed by their personality traits.

**Fig. 1:**
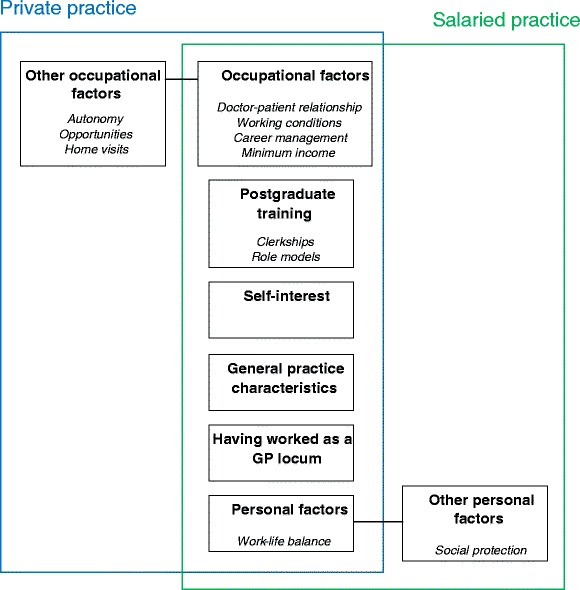
Factors determining choice of practice in young general practitioners

Finally, both salaried and private GPs had common determinants of their choice of practice (Table 3). Many were influenced by occupational factors, especially working conditions. Other specific factors could either be persuasive or dissuasive (Table 4). The characteristics of general practice, the FFS scheme and home visits had dissuaded some young GPs from choosing private practice (Fig. 1) while the idea that they might lose their autonomy was dissuasive to those who preferred private practice.

**Table 3:** Common determinants of choice of private or salaried practice in young GPs

Themes	Factors
Occupational factors	Minimum income, varied scope of practice, focused practice, working time flexibility, part-time work, teamwork, career flexibility
Postgraduate training	Postgraduate clerkships, role models
Having worked as a locum	
Personal factors	Work-life balance, partner’s work, having children, having a doctor as parent
Self-interest	Personality traits, intellectual stimulation, access to new skills and knowledge

**Table 4:** Specific factors determining private or salaried practice

Factors	Private practice	Salaried Practice
Facilitators	Autonomy, closeness in doctor-patient relationship	Social protection
Barriers	Fee-for-service scheme, home visits, workload, provider-client relationship, insufficient learning in private practice and general practice	Hierarchy

## Discussion

Self-interest, occupational factors, postgraduate training and having worked as a locum influenced choice of practice in young GPs. However, these were insufficient. Both salaried and private GPs wanted to find a work-life balance. They also expected flexibility in their working conditions and career management. Young GPs who had little interest in the characteristics of general practice, FFS and home visits eventually went into salaried practice. Income seemed to be a minor criterion of choice.

As in other studies [23–26], working conditions and income were factors that may influence the practice choice of GPs in our study. However, although young GPs thought that income level was perhaps higher in private practice, they above all sought a minimum income. In our study, administrative tasks were more a factor of dissatisfaction than a barrier to choosing private general practice. These results are similar to those in previous studies [23, 24].

Some young GPs wished to develop their careers. Many of them expected to go from private practice to salaried practice later or vice-versa. They sometimes managed to combine both in a mixed practice. General practice trainees perceived career development opportunities as a positive factor in a study by Beaulieu et al [27]. This career flexibility was described as primary care practice “à la carte” by Geneau et al [26]. It could be a way for obtaining varied practice for new GPs, as demonstrated by Abelsen and Olsen [28].

The negative or positive effect of GPs role models on their specialty choice has also already been described [10, 17, 29–32]. Clerkships in GPs’ offices have been described as a factor influencing the career choice of medical students [10, 14, 16, 17, 29, 30, 33–35]. Wesnes et al showed that there was an association between choosing a general practice career and the sum of pre-graduate educational hours regarding general practice or the number of GP teachers [36]. We did not find any previous studies like ours describing the effect of postgraduate training on GPs’ choice of practice.

The characteristics of general practice have been defined by the World Organization of National Colleges, Academies and Academic Associations of General Practitioners/Family Physicians (WONCA) [37]. The varied scope of practice is one of these characteristics that medical students easily identify [32, 34]. The link between the intention to choose general practice and understanding these characteristics is not constant among medical students across the studies [10, 12, 19, 29, 32, 38–40]. This might reflect the lack of knowledge and training in general practice among medical students. It also reflects the need to expose them very early to general practice so that they can understand its characteristics.

Work-life balance seems to be a major factor in choosing general practice among medical students or general practice trainees [16, 27, 30, 39, 41, 42]. As in our study, it was often expected by students and was obtained by those GPs who managed to work shorter or flexible hours [23, 24, 32, 34, 35, 43, 44].

Surprisingly, the profession of the GP’s partner was described as a factor that could influence practice and location. To our knowledge, this has not been reported to date. According to a national report, 59.5 % of French private GPs had a working partner in 2005 [45]. Female and young GPs often had fewer unemployed partners. In most cases, the partner was a senior executive. The need to find a job for the partner and expecting a work-life balance when two young partners work are factors that probably influence young GPs more than their elders.

### Limitations and strengths

The study has some limitations. Participants were recruited from only one region in France (Gironde) and our sample was short. For reasons of feasibility, we redefined private and salaried practice. According to our definitions, eligible young GPs with mixed practice were classified according to the practice which they exercised more. Only one doctor with a mixed practice was included in our sample. We also redefined rural and urban areas. We sampled considering having (or not) additional training to general practice as a major variable. We thought some doctors who decided to undertake additional training were looking for a focused practice. We wanted to know if this changed their choice. However, we did not manage to completely diversify our sample on this variable. Finally, the main limitation of this study is the non-participation of salaried GPs practicing in community health centers. Indeed, there were no young GPs in our area practicing in community health centers. Salaried GPs in community health centers are a minority in France while they represent the main group of GPs in Sweden and Finland [1]. Thus, the majority of French salaried GPs do not practice ambulatory general medicine. For many French postgraduates in general practice, choosing a full-time salaried medical activity could mean refusing to practice (totally or temporarily) ambulatory general medicine. This could explain why some of our doctors who had knowingly chosen to learn general practice as a specialty eventually chose to become salaried doctors. They were unable to identify with the characteristics of general practice and thus rejected them for salaried practice.

We checked that our study was designed in accordance with the COREQ checklist [46]. It conformed with 31 of the 32 criteria. Transcripts were not returned to the participants for their comments.

However, it is one of the first studies to focus on determinants of choice of practice among young GPs in France. We strove to achieve a maximum diversity in our sample during recruitment. We asked all participants for their marital status and number of children so that they could be taken into account when building the sample. None of the doctors we called refused to participate in the study. Although the sample included only young doctors in the South West in France, our results are similar to those found in other countries.

## Conclusions

Young GPs have common determinants of their choice of practice: they expect to find job satisfaction, to work in good conditions and to find a work-life balance. They choose the practice they think is the most appropriate to meet these requirements.

Beyond developing private general practice by financial incentives, all efforts should lead towards the promotion of ambulatory general practice in all its forms. This could be done in several ways: by organizing ambulatory group exercises in multidisciplinary medical homes or community health centers; by reducing paperwork and administrative tasks; by promoting the activities carried out by GPs outside time spent with patients such as teaching, training, prevention, screening and research; by facilitating the flexible and scalable exercise of general practice in terms of career; by diversifying the modes of remuneration of GPs.

A similar study could be led with young doctors in other specialties. This would show whether the factors determining their choices of practice are the same and throw light on what really determines the global primary health care supply in France.

## Abbreviations

FFS: Fee-for-service
GP(s): General practitioner(s)
